# Clinical Outcome of Arthroscopic Remplissage as Augmentation During Arthroscopic Bankart Repair for Recurrent Anterior Shoulder Instability

**DOI:** 10.2174/1874325001711011268

**Published:** 2017-11-10

**Authors:** Ryosuke Miyamoto, Atsushi Yamamoto, Hitoshi Shitara, Tsuyoshi Ichinose, Daisuke Shimoyama, Tsuyoshi Sasaki, Noritaka Hamano, Tsutomu Kobayashi, Toshihisa Osawa, Kenji Takagishi

**Affiliations:** 1 Gunma University Graduate School of Medicine - Orthopaedic Surgery, Maebashi, Gunma, Japan; 2 St. Pierre Hospital - Orthopaedic Surgery, Takasaki, Gunma, Japan; 3 Takasaki University of Health and Welfare - Physical Therapy, Takasaki, Gunma, Japan; 4 National Hospital Organization Takasaki General Medical Center - Orthopaedic Surgery, Takasaki, Gunma, Japan

**Keywords:** Shoulder instability, Bankart lesion, Hill-Sachs lesion, Bankart repair, Remplissage, Arthroscopic repair

## Abstract

**Purpose:**

We aimed to assess functional outcomes and postoperative recurrence rate associated with the remplissage procedure used for bone augmentation with Bankart repair in patients with Hill-Sachs lesions after shoulder dislocation.

**Methods:**

Preoperative computed tomography was performed to check for bony Bankart lesions,calculate the bone defect rate, and estimate the risk for re-dislocation. Functional and clinical scores were assessed preoperatively and at three months, six months, and one year postoperatively.

**Results:**

Between 2011 and 2014, 18 patients (17 male; age at surgery, 29.0±10.4 years; 18 affected shoulders) underwent arthroscopic Bankart repair with arthroscopic remplissage (remplissage group), and 18 sex- and age-matched controls underwent arthroscopic Bankart repair alone (control group). The incidence of bony Bankart lesion and glenoid bone defect was significantly higher in the remplissage group. No complications, re-dislocation, or re-subluxation was noted during or after the operation. Postoperatively, the range of motion and muscular weakness alleviated with time, and the clinical scores improved significantly from the preoperative values. However, the remplissage group showed significantly restricted shoulder flexion, abduction, internal rotation and external rotation even at one year postoperatively. Compared to the control group, the remplissage group showed significantly lower Rowe scores preoperatively, and both Rowe scores and University of California-Los Angeles scale scores remained significantly lower throughout the one-year follow-up.

**Conclusion:**

Despite some restriction of external rotation, remplissage leads to better clinical scores and no recurrence, providing a valid means of augmentation for Bankart repair in high-risk patients with engaged Hill-Sachs lesion.

## 1 BRIEF INTRODUCTION

Bankart repair is designed to repair the labrum-articular capsule complex freed from the anteroinferior aspect of the glenoid (Bankart lesion) after anterior dislocation of the shoulder. In recent years, arthroscopic Bankart repair has become the gold standard to treat shoulder instability [1, 2]. Bankart repair typically achieves shoulder stability and is associated with low postoperative recurrence of dislocation. However, recurrence of shoulder instability has been reported, and high-risk populations include athletes with high activity levels (particularly those involved in collision/contact sports or overhead sports) and patients with large bone defects in the glenoid or the humerus.

Like Bankart lesions, Hill-Sachs lesions also arise from anterior dislocation of the shoulder, but represent compressive injuries to the posterolateral side of the humerus and are commonly found after the first dislocation (67%) [3]. A large Hill-Sachs lesion is a known risk factor for re-dislocation after Bankart repair, and recently developed predictors of re-dislocation risk account for the effect of glenoid bone loss on shoulder instability in the form of the “glenoid track” concept [4-6]. Therefore, bone augmentation seems essential in patients with high risk of re-dislocation if treated with Bankart repair alone. The remplissage procedure, whereby the bone defect is filled by advancing the posterior articular capsule and the infraspinatus muscle tendon, is an augmentation method that may reduce the risk of re-dislocation after Bankart repair in patients with large Hill-Sachs lesions. Remplissage was developed in 1972 by Connolly [7], and was first applied clinically under arthroscopic guidance in 2004 by Purchase *et al.* [8] Several reports have described the outcome of treatment involving remplissage, with favourable outcomes reported even in patients with high risk of instability recurrence expected after Bankart repair alone [9, 10]. However, since this augmentation method is designed for non-anatomical repair, it has been reported to restrict the range of motion, particularly in terms of external rotation. As the outcomes of surgery involving this relatively new procedure have not been sufficiently described to date, there is significant debate over its indications [11, 12].

The present study was undertaken to better assess the functional outcomes and recurrence rate associated with the remplissage procedure. For this purpose, we compared the outcome of repair for anterior shoulder instability between patients undergoing arthroscopic Bankart repair alone and those undergoing arthroscopic Bankart repair combined with arthroscopic remplissage augmentation. The hypothesis tested in this study was that arthroscopic remplissage as a means of bone augmentation during arthroscopic Bankart repair in high-risk patients with anterior shoulder instability can reduce the postoperative re-dislocation rate without restricting the range of motion (ROM) or reducing clinical scores.

## 2 MATERIALS AND METHODS

### 2.1 Subject

The study involved patients who underwent arthroscopic Bankart repair (alone or with arthroscopic remplissage) at our facility between April 2011 and March 2014. At our facility, the indications for remplissage are: (1) Hill-Sachs lesion and high risk for postoperative re-dislocation (*e.g.*, when the patient’s profession involves collision/contact sports), and (2) intraoperative finding of engaged Hill-Sachs lesion [4]. A total of 18 patients (17 male, one female; age at surgery, 29.0±10.4 years; 18 affected shoulders) underwent Bankart repair with remplissage. The control group consisted of 18 age- and sex-matched patients who underwent arthroscopic Bankart repair alone (*i.e.*, without remplissage) during the same period. The study was conducted with the approval of the ethics review board of our facility, and written consent was obtained from each patient prior to inclusion in the study.

### 2.2 Surgical Technique

Surgery was performed by one of three shoulder surgeons (A.Y., H.S., T.K.), according to the same operative procedure, with the patient in the beach-chair position. An arm positioner attached to the affected arm was used to freely adjust the position of the affected arm as necessary. General anaesthesia was applied in combination with interscalene block. First, an arthroscope was inserted *via* a routine posterior portal for evaluation of Bankart and Hill-Sachs lesions. The presence/absence of engaged Hill-Sachs lesion was evaluated in accordance with the method described by Burkhart *et al.* [4]

In patients who received remplissage, the arm underwent specific manipulation prior to initiating the procedure. Under posterior arthroscopic guidance, the affected arm was placed in the position of abduction and external rotation, and the bone head was pushed forwards, to make observation and manipulation of the Hill-Sachs lesion easier. A spinal needle was inserted at a point close to the posterior angle of the acromion, and the position of the remplissage portal was decided so that a suture anchor could be later inserted at an optimum angle. Then, the deltoid muscle and the tendon plate were perforated to create a portal. A cannula 5 mm in diameter (Smith & Nephew Endoscopy, Mansfield, MA, USA) was kept in the remplissage portal. A bar was then inserted *via* the remplissage portal, followed by refreshing and decertification of the Hill-Sachs lesion. Subsequently, a double-loaded suture anchor (GRYPHON BR; DePuy Mitek, Raynham, MA, USA) was inserted *via* the remplissage portal, and two anchors (one above and the other below the Hill-Sachs lesion) were set. The arthroscope was dislocated into the subacromial space and the tendinous elements of the infraspinatus muscle and the teres minor muscle were observed directly (bursoscopy). While moving the arthroscope appropriately between the gleno-humeral joint and the subacromial space, suture replacement was performed using a 60° Suture Grasper (DePuy Mitek). Suture replacement was carried out in such a way as to fix only the tendinous elements of the infraspinatus and teres minor, to avoid postoperative restriction of ROM, with mattress suture applied to two sites for each anchor.

Manipulation related to remplissage was suspended in this step, and ordinary Bankart repair was performed using four suture anchors (GRYPHON BR; DePuy Mitek), in accordance with the method described by Sugaya *et al.* [13] Afterwards, the arthroscope was inserted into the subacromial space, and knot tying of the remplissage suture was performed with the shoulder in maximum external rotation, to avoid potential restriction of the ROM of external rotation. Upon completion of all manipulations, the arthroscope was inserted *via* the anterior portal to confirm appropriate augmentation and to complete the operation.

### 2.3 Postoperative Protocol

All patients received postoperative care according to the same protocol, regardless of whether or not remplissage had been performed. On the day following the operation, a shoulder immobilization device (DonJoy Ultrasling II^®^; DJO Global, Vista, CA, USA) was applied, and passive ROM training was started to an extent that there was no pain. Three weeks after the operation, the immobilization device was removed, and active exercise was permitted. Strengthening exercises of the rotator cuff and scapular stabilizers were started six weeks after the operation. Three months after the operation, the patients were permitted to resume sport activities, excluding contact play and throwing. Six months or more after the operation, the patients were permitted to resume all sport activities, depending on the course of functional recovery.

### 2.4 Evaluation Items

Preoperatively, a computed tomography scan of the bilateral shoulder joints was performed to check for bony Bankart lesions and calculate the bone defect rate (ratio between the transverse dimension on the affected side and the transverse dimension on the intact side). In addition, using the method described by Yamamoto *et al.* [6], the glenoid track was measured to judge whether the lesion can be considered “off-track”, which is considered to involve a high risk for re-dislocation. Immediately before and at three months, six months, and one year after the operation, the shoulder joint ROM (flexion, abduction, external rotation, internal rotation) and shoulder joint muscle strength (abduction, external rotation) were measured as functional parameters. Pain on the visual analog scale (VAS), the Rowe score, and the University of California–Los Angeles (UCLA) Shoulder Rating Scale score were assessed as clinical scores. The shoulder joint ROM was measured using a goniometer, with the patient in a sitting position. Flexion and abduction were measured as the angle formed by the trunk and the humerus during anterior and lateral raising of the arm, respectively. External rotation was measured as the angle formed by the sagittal plane of the trunk and the forearm during 90° flexion of the elbow, with the arm hanging down. Internal rotation was measured as the highest level of the vertebral spinous process that the patient could reach with the thumb when placing the dorsum manus on the back and moving the thumb cranially. For the purpose of statistical analysis, internal rotation was recorded as the following numbers: 1 for the first thoracic vertebra, 1 through 12 for the first to the twelfth thoracic vertebra, 13 through 17 for the first to the fifth lumbar vertebra, 18 for the sacrum, 19 for the buttocks, and 20 for the thigh. Shoulder joint muscle strength was measured using a wireless digital handheld dynamometer (micro FET2^®^; Hoggan Scientific, West Jordan, UT, USA), with the patient in the sitting position. Abductor strength and external rotator strength were measured as the isometric muscle strength under the following conditions: (1) for measuring abductor muscle strength, with the palm placed down and the shoulder joint in 90° abduction; (2) for measuring external rotator muscle strength, with the arm hanging down, the shoulder joint in a position intermediate between internal and external rotation, and the elbow joint in 90° flexion. Each measurement of muscle strength was performed in triplicate, and the median was retained. To ensure reliability, evaluation of functional outcomes and clinical scores was carried out by an independent examiner blinded as to the operative procedure.

### 2.5 Statistical Analysis

The study population was divided into two groups: the remplissage group, which involved patients who underwent arthroscopic remplissage in addition to arthroscopic Bankart repair, and the control group, which included patients who underwent arthroscopic Bankart repair alone (*i.e.*, without remplissage). The values of preoperative parameters were compared between the two groups using the Chi-square test and unpaired t-test for categorical variables and continuous variables, respectively. The values of parameters recorded at four time points (preoperatively and at three months, six months, and one year postoperatively) were evaluated within each group and compared by means of repeated-measure analysis of variance. Any parameter found by this analysis to vary significantly was further subjected to a post-hoc test with Bonferroni correction. Outcomes were compared between the two groups using the unpaired t-test. Statistical analyses were performed using IBM SPSS Statistics version 21 (IBM Corp., Armonk, NY, USA). P-values <0.05 were regarded to indicate statistical significance.

## 3 RESULTS

No significant difference between the remplissage and control groups was noted in terms of age, male-to-female ratio, affected side, preoperative ROM, or preoperative muscle strength Table (**1**). However, the preoperative Rowe score was significantly poorer in the remplissage group, reflecting that this group consisted mostly of high-risk patients. The incidence of bony Bankart lesion and glenoid bone defect was also significantly higher in the remplissage group.

No complications were noted during or after the operation. Over a follow-up of one year, no re-dislocation or re-subluxation was noted. Postoperatively, the restricted ROM and muscular weakness alleviated with time, and the clinical scores improved significantly from the preoperative values Table (**2**). However, significant restriction of external rotation remained even at one year after the operation in the remplissage group. No significant difference in postoperative scores was noted among cases managed by different surgeons (data not shown).

While there was no difference in preoperative ROM between the two groups, at one year after the operation, the ROM of flexion, abduction, external rotation, and internal rotation was significantly lower in the remplissage group Tables (**2** and **3**). Muscle strength did not differ between the two groups before or after the operation. Of the clinical scores, the VAS score differed significantly between the two groups at six months after the operation but not at one year postoperatively. The Rowe score was significantly lower in the remplissage group both preoperatively and at one year after the operation. The UCLA scale score remained significantly different between the two groups throughout the follow-up period.

## 4 DISCUSSION

Burkhart *et al.* reported that recurrence after Bankart repair alone was seen in 21 (10.8%) of 194 patients; among the 21 patients with recurrence, 7 were free of significant bone defect, but 14 had a large Hill-Sachs lesion, suggesting the significance of this lesion as a risk factor for re-dislocation after Bankart repair [4]. A high incidence of re-dislocation was also reported among athletes involved in collision/contact sports [14-16]. Yamamoto *et al.* reported that the re-dislocation rate following arthroscopic Bankart repair was three times higher among patients involved in contact sports than among those not involved in contact sports, and that only 24% of athletes recovered to pre-injury activity levels, because of residual instability and ROM restriction [9]. Remplissage is recently becoming more popular for the management of such high-risk cases. The Latarjet procedure, which involves transplantation of the coracoid process into the glenoid together with a common tendon, is also applicable in such patients. While the postoperative recurrence rate does not differ between the Latarjet and remplissage procedures, a higher incidence of complications such as nerve injury, bone graft dislocation, and bone nonunion was reported for the Latarjet procedure [12, 17, 18]. In addition to the lower rate of complications, remplissage has the advantage that it can be performed under arthroscopic guidance during Bankart repair [10, 12]. Buza *et al.* reported a mean recurrence rate of 5.4% (9/167 shoulders) at an average of 26.8 months after remplissage [19], while Garcia *et al.* reported a recurrence of 11.8% (6/51 shoulders) at an average of 60.7 months after remplissage [20]. Based on these previous findings, we indicated remplissage as a means of augmentation for Bankart repair in high-risk patients (*e.g.*, patients involved in collision/contact sports) and in patients with intraoperative findings of engaged Hill-Sachs lesions. We noted no re-dislocation during the first year postoperatively, which confirms that remplissage is a valid means of augmentation for Bankart repair in high-risk patients and in patients with engaged Hill-Sachs lesions.

Restriction of ROM represents a shortcoming of remplissage, as described in many reports, and it results from the fact that remplissage is a technique of non-anatomical repair whereby the Hill-Sachs lesion is filled by advancing the posterior articular capsule and the infraspinatus muscle tendon; the new footprint is located closer to the posterior articular cavity edge, which can cause ROM restriction (particularly restriction of external rotation). The biomechanical study of Grimberg *et al.* found that patients who underwent remplissage showed an 11.7° restriction of external rotation ROM, which differed significantly from that noted in patients who underwent Bankart repair alone [21]. Zhu *et al.* reported an average improvement in flexion by 8° and restriction of external rotation by 1.9°, but these differences were not statistically significant [22]. Boileau *et al.* reported that, compared to the intact side, the side treated with remplissage showed significant restriction of flexion, external rotation, and internal rotation at six months postoperatively and at the end of follow-up (24 months on average), with an external rotation restriction of 14±14° at six months and 8±7° at the end of follow-up [23]. To avoid postoperative ROM restriction, Cho *et al.* modified the operative procedure by skipping infraspinatus muscle tendon fixation and applying only posterior articular capsule fixation; however, at the end of the follow-up, they found restriction of external rotation by 8±23° compared to the value for external rotation on the intact side [24]. In the present study, we attempted to avoid postoperative ROM restriction by directly inspecting the infraspinatus and teres minor tendons through the subacromial space and fixing the tendinous elements instead of the muscles in a reliable manner. However, similar to Cho *et al.* we found significant restriction of external rotation at one year after the operation, and significant restriction of flexion, abduction, and external rotation compared to the values noted in the control group. Augmentation with remplissage is quite useful in preventing the recurrence of instability after surgery, but its application needs to be judged carefully in view of the potential of postoperative restriction of ROM (particularly ROM of external rotation).

As another shortcoming of remplissage, persistent postoperative pain has been reported sporadically. Nourissat *et al.* reported that 5 of 15 patients complained of pain at the end of follow-up, at an average of 27 months after remplissage; however, none of the 17 patients in the control group (who had undergone Bankart repair alone) complained of pain. Of the 5 patients complaining of pain, 3 had pain during abduction/external rotation, suggesting the presence of posterior impingement by the posterior labrum and the new footprint [25]. Lädermann *et al.* evaluated the outcomes of remplissage in a cadaveric study using fresh-frozen shoulder specimens, and reported that capsulomyodesis rather than capsulotenodesis was often noted, reflecting the outbreak of muscle strangulation due to remplissage [26]. Such strangulation of the muscle may indeed lead to pain. In the present study, we modified the operative procedure to avoid muscle strangulation. Indeed, while the VAS score was significantly different between the two groups at six months after the operation, it improved thereafter, showing no significant difference between the groups at one year postoperatively. Persistence of pain, as reported by Nourissat *et al.* was not seen in the patients included in the present study, suggesting that the modification of the operative technique adopted in the present study is likely effective in preventing residual pain.

Limitations of the present study include the small sample size and short follow-up period. Additionally, we did not consider the role of surgeon-specific techniques, the influence of the size of the Hill-Sachs lesion, or the extent of recovery of sports performance. Furthermore, the present study involved patients with various risk for postoperative re-dislocation; further study involving a sample of high-risk patients only is warranted to evaluate the outcome of the operative procedure in this special population.

## CONCLUSION

The present study found clear evidence that remplissage leads to better outcomes in terms of clinical scores and lack of recurrence, despite some restriction of external rotation. Therefore, our findings confirm the validity of remplissage as a means of augmentation for Bankart repair in high-risk patients and in patients engaged Hill-Sachs lesions.

## Figures and Tables

**Table 1:** Preoperative characteristics of remplissage group and control group.

	Remplissage Group (n=18)	Control Group (n=18)	p-value
Background factor			
Age	29.0 ± 10.4	27.8 ± 11.2	0.739
Gender	17 / 1	17 / 1	1.000
(male / female)			
Surgery on the dominant side	9 / 9	10 / 8	1.000
(dominant / non-dominant)			
Range of motion (deg.)			
Flexion	156.1 ± 9.6	161.4 ± 11.4	0.219
Abduction	160.9 ± 20.1	168.1 ± 15.8	0.251
External rotation	59.4 ± 12.5	59.4 ± 14.3	0.901
Internal rotation	8.4 ± 2.1	7.6 ± 2.0	0.228
Muscular power (Kg)			
Abduction	7.7 ± 2.1	6.8 ± 1.3	0.084
External rotation	7.7 ± 2.3	7.0 ± 1.1	0.167
Clinical score			
VAS	19.2 ± 24.0	11.3 ± 15.8	0.255
Rowe score	27.7 ± 7.3	35.6 ± 9.5	0.019*
UCLA scale	25.6 ± 2.3	25.3 ± 3.0	0.713
Bone defect rate of glenoid (%)	13.0 ± 5.6	7.3 ± 4.7	0.004*
Bony bankart lesion	9	2	0.004*
Glenoid track (off-track)	12	0	-

VAS: Visual analogue scale at movement

*indicates statistical difference compared between two groups (p < 0.05)

**Table 2:** Postoperative outcomes of remplissage group and control group.

	Preop.	Postop.3Mo	Postop.6Mo	Postop. 1 yr
**Remplissage group**				
Range of motion (deg.)				
Flexion	156.1 ± 9.6	129.7 ± 23.2*	143.8 ± 17.2*	155.0 ± 11.1
Abduction	160.9 ± 20.1	126.2 ± 26.3*	146.8 ± 20.0	158.0 ± 12.1
External rotation	59.4 ± 12.5	32.9 ± 18.9*	42.1 ± 15.2*	44.5 ± 11.7*
Internal rotation	8.4 ± 2.1	11.2 ± 3.3*	9.9 ± 2.7	8.6 ± 2.3
Muscular power (Kg)				
Abduction	7.7 ± 2.1	4.3 ± 1.9*	7.3 ± 2.6	7.5 ± 2.7
External rotation	7.7 ± 2.3	5.5 ± 2.2	7.1 ± 2.0	7.0 ± 2.6
Clinical score				
VAS	19.2 ± 24.0	14.4 ± 18.1	18.8 ± 18.8	6.2 ± 8.6
Rowe score	27.7 ± 7.3	69.7 ± 17.9*	88.5 ± 8.8*	88.5 ± 8.8*
UCLA scale	25.6 ± 2.3	23.3 ± 6.1	27.9 ± 5.3	32.9 ± 1.9*
**Control group**				
Range of motion (deg.)				
Flexion	161.4 ± 11.4	145.8 ± 15.3*	158.9 ± 9.8	165.0 ± 6.2
Abduction	168.1 ± 15.8	148.8 ± 24.9*	162.8 ± 11.7	171.7 ± 6.4
External rotation	59.4 ± 14.3	45.8 ± 16.6	56.4 ± 12.0	65.0 ± 9.7
Internal rotation	7.6 ± 2.0	9.4 ± 2.1*	7.9 ± 1.2	6.8 ± 1.1
Muscular power (Kg)				
Abduction	6.8 ± 1.3	4.8 ± 2.4*	6.5 ± 2.0	8.1 ± 1.4*
External rotation	7.0 ± 1.1	6.9 ± 1.8	7.2 ± 1.9	8.5 ± 1.9
Clinical score				
VAS	11.3 ± 15.8	11.2 ± 17.3	2.7 ± 7.0	1.4 ± 4.4
Rowe score	35.6 ± 9.5	80.3 ± 11.0*	90.6 ± 8.4*	98.9 ± 2.1*
UCLA scale	25.3 ± 3.0	27.9 ± 4.9	33.6 ± 2.2*	34.8 ± 0.6*

Preop.: Preoperative, Postop.: Postoperative, VAS: Visual analog scale at movement

*indicates statistical difference compared to preoperative (p < 0.05)

**Table 3:** Comparison of the postoperative outocomes between remplissage group and control group.

	p-value			
	Preop.	Postop. 3Mo	Postop. 6Mo	Postop. 1 yr
Range of motion				
Flexion	0.243	0.020*	0.004*	0.021*
Abduction	0.320	0.036*	0.006*	0.001*
External rotation	0.389	0.039*	0.004*	0.000*
Internal rotation	0.179	0.068	0.010*	0.039*
Muscular power				
Abduction	0.144	0.600	0.354	0.378
External rotation	0.178	0.058	0.951	0.085
Clinical score				
VAS	0.178	0.628	0.005*	0.107
Rowe score	0.008*	0.054	0.020*	0.005*
UCLA scale	0.720	0.029*	0.001*	0.008*

Preop.: Preoperative, Postop.: Postoperative, VAS：Visual analog scale at movement

*indicates statistical difference (p < 0.05)
